# It is not just about “the trial”: the critical role of effective engagement and participatory practices for moving the HIV research field forward

**DOI:** 10.1002/jia2.25179

**Published:** 2018-10-18

**Authors:** Kathleen M MacQueen, Judith D Auerbach

**Affiliations:** ^1^ Global Health Research FHI 360 Durham NC USA; ^2^ Department of Medicine University of California San Francisco CA USA

**Keywords:** community engagement, stakeholder engagement, participatory research

Engagement by and with affected people, communities, and other stakeholders has been a critical part of HIV treatment and prevention research since the earliest days of the epidemic 1, 2. It has been a force for moving important research forward through advocacy, as well as, a disrupter of research and its translation to practice when inadequate engagement creates possibilities of exploitation. Over the decades, there has been a gradual accumulation of experience on what engagement means, how to effectively engage diverse stakeholders, and how context influences the effectiveness of different engagement practices. We have also seen a gradual progression from engagement mainly as a consultative mechanism towards a fuller use of participatory practices. Advocates have led the way by creating independent structures such as the AIDS Coalition to Unleash Power (ACT UP) in 1987, Treatment Action Group (TAG) in 1992, AIDS Vaccine Advocacy Coalition (AVAC) in 1995, and the Alliance for Microbicides and the Global Campaign for Microbicides in 1998, as well as by pushing for leadership structures within major funding networks, such as the Community Partners 3 and the Legacy Project within the NIH Office of HIV/AIDS Network Coordination (HANC). Research advocacy organizations continue to emerge such as the New HIV Vaccine and Microbicide Advocacy Society (NHVMAS) in Nigeria, Africa free of New HIV Infections (AfNHI), and the International Rectal Microbicide Advocates (IRMA).

In parallel with this advocacy movement within HIV research, bioethicists historically have been engaged in a broader global discussion of the role of communities in research. UNAIDS called for the involvement of community representatives “in an early and sustained manner” in HIV vaccine trials in 2000 4, and placed increased emphasis on community participation in guidance for biomedical HIV prevention trials more broadly in 2007 5. The Council for International Organizations of Medical Sciences (CIOMS) provides detailed commentary on the need to engage research participants and communities “in a meaningful participatory process that involves them in an early and sustained manner” 6. The National Health Research Ethics Council of South Africa recommends similar engagement by communities for health research generally and requires it for population‐focused HIV prevention research specifically 7.

While the importance and legitimacy of engaged and participatory practices increasingly is recognized as a vital component of HIV and other health research, it nonetheless remains largely compartmentalized within the scientific process. For example, in many HIV research networks, community representation is mandated on protocol teams and implementing sites are required to have community advisory boards (CABs) or similar mechanisms in place, but representatives and CABs are not resourced or structurally supported in ways that parallel the contributions of laboratories, biostatistics, and clinical components. Protocol teams struggle to balance calls for substantive community participation in the early stages of research development and the pressure from funders to minimize costs and timelines to implementation. Advocates raise concerns that engagement practices are in danger of being reduced to window dressing, while researchers and funders raise equally important questions about the evidence that the time and resources invested in engagement ultimately enhance the ethical and scientific outcomes of the research. Systematic evaluation could assure advocates, researchers, and funders of the quality and value of engagement, yet it is rare. In fact, while the practice of engaged research has proliferated the science of it still is in early development 8, 9, 10, 11, 12.

Creating an evidence base for community and stakeholder engagement in HIV‐related research is not an easy task. The importance of understanding what will work, for whom, and under what conditions has increased as the boundaries between HIV prevention, treatment, and cure research have blurred and intervention strategies have become ever‐more technological and differentiated 13. This heightens the need to attend to context and its multiple dimensions–culture, politics, religion, history, economics, gender, family/kinship systems, and social hierarchies of race and ethnicity–in tandem with whatever specific HIV strategy is being explored.

While social scientists always argued for the need to pay attention to context, this generally fell on deaf ears in the biomedical HIV clinical trials world, until a few big controversies erupted in the 1990s and early 2000s. In 1994, the ACTG 076 trial demonstrated that AZT, the only retroviral treatment available at that time, was effective for preventing HIV transmission in utero and during birth 14. The treatment regimen used in the trial was expensive, complex, and clinically demanding. Concerned that the treatment could therefore not be used in lower‐middle income country (LMIC) settings where mother‐to‐child transmission rates were highest, a global effort began to field trials to test effectiveness of less intensive treatment regimens with a greater potential for scale‐up in those settings. The trials were designed to compare the experimental regimens against a placebo, on the grounds that this reflected the current standard of care. The argument was that use of the ACTG 076 regimen as a comparator could result in rejection of effective new regimens that fell short of the ACTG 076 standard, and that such a design would also require both more resources and more time to implement 15. As some analysts have argued, the trial design highlighted and sought to address global health inequities at one level, but failed to challenge them at another 16. The trials raised important ethical questions about higher‐income country (HIC) researchers’ responsibilities to LMIC trial participants who lack access to effective interventions and treatment. The controversies led to revisions in international ethics guidelines, setting the stage for ongoing debates about the appropriate use of local versus global standards of care in the design of ethical trials, and who gets to decide what is appropriate and what is exploitative 17, 18, 19, 20.

The controversial trials of simplified regimens to reduce mother‐to‐child HIV transmission were completed, despite the controversies, and showed efficacy. This led many HIV researchers to feel validated in their view of what constituted an appropriate balance between science and ethics in a research design. But for many advocates, the trials were just one more brick in a wall of global inequity that they were determined to tear down. By the early 2000s AIDS treatment activists built high‐level support to expand antiretroviral treatment globally, despite widespread scepticism that such programmes could be successfully implemented in economically disadvantaged countries 21. At the same time, the first pre‐exposure prophylaxis (PrEP) trials were being planned in Cambodia and West Africa, but not in coordination with the effort to expand global treatment. For some treatment advocates, this raised several red flags: Why would researchers test a new, and at that time very expensive, antiretroviral drug in settings with limited access to similar drugs for treatment? Were poor women in poor countries being exploited, so that a drug company could profit from the sale of PrEP in rich countries? If antiretrovirals could be made available for a prevention trial, why couldn't participants who became infected in that trial then be provided antiretroviral treatment aligned with global standards 22, 23, 24? This new round of controversies led to the closure of a PrEP trial in Cameroon and prevented implementation of another in Cambodia 23. Following this disruption, a three‐year effort ensued during which UNAIDS, civil society representatives, advocates, researchers, funders, and bioethicists came together in a series of meetings that culminated in the creation of Good Participatory Practice (GPP) for HIV Prevention Trials, to parallel existing practice guidelines for clinical, laboratory, and epidemiological research 25, 26.

This special supplement aims to explore the impact of GPP and broader community engagement efforts on the conduct and outcomes of HIV (chiefly prevention) research. We begin by looking at the state of engagement practice today. Day and colleagues conducted a scoping review of community engagement in HIV clinical trials, using benchmarks outlined in GPP guidelines: the variety of stakeholder engagement methods used, the variety of types of stakeholders engaged, and how engagement aligned with all stages of clinical research (pre‐trial, implementation, and post‐trial) 27. The results are encouraging in that the benchmarks were met to at least some degree by all of the 108 studies included in the analysis. However, the authors found that many benchmarks were met using researcher‐driven methods such as focus groups and interviews, rather than true participatory processes. They also found fewer studies reporting stakeholder engagement in LMIC than other income‐status countries, and a general tendency to focus engagement on the early stages of trial planning rather than all along the trial's trajectory.

A challenge faced by Day and colleagues in their analysis is the fact that no standards exist for reporting on stakeholder engagement related to HIV (or other) clinical trials. Clinical triallists are fond of saying that “if it isn't documented, it didn't happen.” The absence of documentation about stakeholder engagement efforts severely limits the systematic accumulation of knowledge and, therefore, opportunities to move the field forward. One option for both assuring a minimal standard for engagement and documenting the elements of that standard is regulatory oversight, as outlined by Slack and colleagues in this issue 28. They describe consensus among extant guidelines that research ethics committees should review engagement for HIV prevention trials, but they note that there is a lack of consensus on what constitutes standards of excellence. At the same time, they note that regulatory oversight requires a delicate balancing act between ensuring compliance and respecting the need for research teams to maintain flexibility and responsiveness in their engagement practices. They argue that inclusion of engagement as part of the ethics review process should not result in a need for approval of amendments to the protocol that would undermine the concept of dynamic responsiveness.

Another aspect of community and stakeholder engagement that has received little attention in the literature is the set of challenges faced by research sites conducting multiple trials with multiple sponsors or other partners. Baron and colleagues present a unique case study highlighting lessons learned from a leading South African research institute in this regard 29. Their analysis goes beyond assessing GPP implementation in the context of a single clinical trial, and documents the experience of implementing it on an institution‐wide level. They also attend to the impact of environmental factors beyond the control of the clinical trial team – in this case, the outcomes of two other trials in the area—on GPP implementation. Through self‐reflection, the authors identify challenges, describe the long‐term problem‐solving strategies undertaken, and provide rich documentation about engagement that likely will prove useful to others.

Case examples and systematic reviews such as those described above are important contributions to building the evidence base for community and stakeholder engagement in HIV research. A persistent gap, however, centres on the need for generalizable data derived from the engagement experiences of multiple communities, research sites and clinical trials. The article by MacQueen and colleagues describes ongoing work aimed at filling this gap 30. While focused on the example of GPP in the context of TB clinical trials, the process outlined by the authors for developing systematic measures is equally informative for the HIV research context. The article highlights the importance of developing a theory‐based framework for evaluation of engagement practices, clarifying the goals of engagement, and engaging stakeholders in an iterative, participatory process to refine the measurement strategy.

Many of the challenges and gaps noted thus far reflect the outlier status of community and stakeholder engagement, that is, that it often is treated as ancillary to trials rather than as an integral dimension on par with clinical, laboratory, regulatory, and statistical components. This problem of viewing engagement narrowly as a tool or mechanism for supporting clinical trials has deeper implications. Pantelic and colleagues argue that engagement should not be viewed as a *method*, but rather as an *orientation* that should be built into the interventions being designed and tested 31. They make the case for shifting the nature and orientation of HIV prevention research to be more aligned with the interests and needs of individuals and for addressing structural barriers to enhancing and integrating knowledge about community engagement in research through community‐based participatory and person‐centred research approaches.

Taking this mindset further, Wheeler and colleagues describe an HIV prevention study built on a long‐term partnership among Black men who have sex with men (BMSM) communities and organizations in the United States, which included a multidisciplinary, multiracial research team led by BMSM together with a Black Caucus, comprised of highly respected multidisciplinary Black professionals 32. The study, a PrEP demonstration project, included community consultations at all sites and staff trainings aligned with the person‐centred research approach. The success of this project underscores the multiple layers of leadership and inclusion—from the grassroots to the institutional to the national—needed for true and effective engagement in communities where there are deep, persistent disparities in HIV and its syndemic co‐travellers by race, ethnicity, and geography. This requires investing resources and building capacity, infrastructure, and scientific leadership within these communities to ensure substantive decision‐making power is available to individuals from those communities who understand how these disparities are experienced.

As HIV research increasingly expands into the area of pragmatic and community‐level interventions, the importance of engagement as more than an ancillary tool becomes even greater. Camlin and colleagues make the point that qualitative research methods can be an important “listening tool” in the engagement process for large‐scale clinical trials and can facilitate meaningful, productive dialogue that enhances intervention design and study implementation 33. In one example, they describe uncovering gender differences in accessing HIV testing services from such qualitative research, and how the presentation of these findings to the clinical research team led to adjustments in the testing campaign. In another, they describe how ongoing qualitative research led to a deeper understanding of the impact of the intervention on the community which led to unanticipated positive social change that fuelled intervention effectiveness. This kind of finding would not be evident from a “typical,” quantitative clinical trial outcome analysis, and it has implications for future community‐level trial design.

Lippman and colleagues carry this theme further in their description of theory‐based community mobilization to reduce HIV acquisition among adolescent girls and young women (AGYW) in sub‐Saharan Africa, where engagement and participatory practice were the intervention rather than merely the means for facilitating interventional research 34. Their study is the first to show that community mobilization is associated with lower HIV incidence among AGYW. In highlighting the components of community mobilization that are protective—including, critical consciousness, leadership, social cohesion, and shared concerns around HIV—the authors point to social‐level factors that can be harnessed for meaningful community engagement in HIV research, and, more importantly, for addressing fundamental inequities and disparities to better combat HIV and other health threats altogether.

## Conclusion

Clinical research is essential, challenging work that has brought us to a point where we can envision a world without HIV. But clinical research alone will not create that world. HIV is a disease that travels with stigma, disparity, and discrimination—social processes that unintentionally may be reproduced in the context of clinical research if appropriate engagement of stakeholders does not occur. The realization of a world without HIV will require political will, social support, and funding, to translate science into the day‐to‐day lives of people and communities, and to have the day‐to‐day realities of people inform science. Experience has taught us that this bi‐directional translation must include stakeholders at all levels, from the streets to global board rooms, and across all stages of research, from the earliest concepts to demonstration projects and programme scale‐up. Stakeholder and community engagement must be fully and systematically integrated into HIV clinical research, and the evidence of its contributions and effectiveness must move beyond anecdotal reporting.

## Competing interests

The authors have no competing interests to declare.

## Authors’ contributions

K.M.M. and J.D.A. wrote the paper. Both authors read and approved the final manuscript.
